# Recent research into healthcare professions regulation: a rapid evidence assessment

**DOI:** 10.1186/s12913-021-06946-8

**Published:** 2021-09-08

**Authors:** Julie Browne, Alison Bullock, Chiara Poletti, Dorottya Cserző

**Affiliations:** 1 Cardiff University, Cardiff Unit for Research and Evaluation in Medical and Dental Education (CUREMeDE), 10/12 Museum Place, Cardiff, CF10 3BG UK; 2 Cardiff University, Cardiff University School of Medicine, Centre for Medical Education, Neuadd Meirionnydd, Heath Park, Cardiff, CF14 4YS UK

**Keywords:** Regulation, Rapid evidence assessment, Review, Patient safety, Fitness to practise, Quality assurance, Registration, Guidelines, Standards

## Abstract

**Background and aims:**

Over the last decade, regulators have taken significant steps towards tackling perceptions that regulatory systems are burdensome. There has been much international research activity in the regulation of health and care professionals.

This article reports a review of studies on health professions regulation between January 2011 and March 2020. Its chief object was to provide robust and up-to-date evidence to assist regulators in policy development and implementation.

The main objectives of this study were to:

1. Identify and retrieve research in the field of health and care professions regulation in English since 2011;
2. Evaluate the published research, exploring its utility to regulators and practitioners, and drawing out any key messages;
3. Draw conclusions concerning the scope and limitations of the research literature and identify areas for further research.

**Methods:**

We undertook a rapid evidence assessment (REA) of the international literature on health and care professions regulation, including reviewing ten UK regulators’ websites to identify issues of concern and strategic priorities. We retrieved 3833 references, using a four-stage screening process to select the 81 most relevant.

**Results:**

Results are reported within six key themes: harm prevention and patient safety; fitness to practise; quality assurance of education and training; registration including maintenance of registers; guidelines and standards and relations with regulatory bodies.

**Conclusions:**

Regulation of professionals in health and care is comparatively undeveloped as a field of academic study. Consequently, the published evidence is diffuse and small-scale. Most work presents relatively weak data of low relevance to regulators, mainly reporting or describing the current position. Few studies are able to show the impact of regulation or demonstrate a causal link between regulation and its effects. To inform their research and policy agendas health and social care regulators need to commission, interpret and apply the scholarly literature more effectively; academics need to engage with regulators to ensure that their research provides high-quality evidence with practical relevance to the regulators’ agendas. Further study is needed to explore how effective academic collaborations between regulators and researchers may be created and sustained.

**Supplementary Information:**

The online version contains supplementary material available at 10.1186/s12913-021-06946-8.

## Background

A 2011 scoping review, exploring the academic literature on the behavioural effects of regulatory activity and interventions on those regulated, concluded that the evidence concerning how professional regulation affects behaviour was both sparse and weak [1]. Since publication of the Quick report, there have been significant changes in how the public and the professions view the function, purpose and effectiveness regulation within the health and care sectors.

The 2011 call for stronger evidence on the effectiveness of professional regulation in ensuring high quality care has been taken up more widely following a number of high-profile systematic failings [2, 3]. Regulators have taken significant steps towards tackling perceptions that their regulatory systems are complicated, and that their overriding culture is punitive and overly concerned with fitness to practise (FtP) by developing more collaborative approaches to regulation based on partnership, open consultation and dialogue [4, 5]. Regulators have become more aware of the need to involve practitioners as a way of increasing trust and confidence and of addressing increased pressures relating to FtP such as higher caseloads and evidence of practitioner concern around FtP [6, 7]. Increasing numbers of documents offering guidance supplementary to professional standards have been produced. Regulators in health and care professions have themselves become more active in generating and using data to inform their work since 2011 [8], and significant quantities of data have been generated during that time.

In this article we contribute to the existing literature base by providing a review of studies on health professions regulation between January 2011 and March 2020. Its chief aim was to provide robust and up-to-date evidence to inform the work of UK regulators in commissioning, analysing, interpreting and using research data to support policy development and implementation.

The main objectives of this study were to:

1. Identify and retrieve research in the field of health and care professions regulation in English since 2011;
2. Evaluate the published research, exploring its utility to regulators and practitioners, and drawing out any key messages;
3. Draw conclusions concerning the scope and limitations of the research literature in health and care professions regulation and identify areas for further research.

Our definition of research in the context of this review was a broad one: we included surveys, consultations, and strategic reviews as well as literature reviews, peer reviewed publications and commissioned research.

## Methods

In this paper we report a desk-based rapid evidence assessment (REA) of the international literature on health and care professional regulation. We also reviewed ten UK regulators’ websites, analysing the most recent annual reports to identify issues of current concern and details of future strategic priorities to gain insights into whether, and if so, how far, the published research literature informed the regulators’ agendas and vice versa. This REA was the central part of a wider study, commissioned by the UK Professional Standards Authority for Health and Social Care (PSA). The School of Social Sciences’ Research Ethics Committee at Cardiff University gave ethical approval for this study.

REAs allow a rapid survey of the extent and quality of literature on a specific issue, permitting any gaps in the evidence to be identified. REAs are therefore ideal for identifying pragmatic approaches to future development. While rapid reviews are not as exhaustive a systematic reviews [9] they permit a systematic approach to searching and evaluating extensive bodies of evidence. The Government Social Research Service’s Rapid Evidence Assessment toolkit was used as the basis for the methods we used [10].

**Fig. 1:**
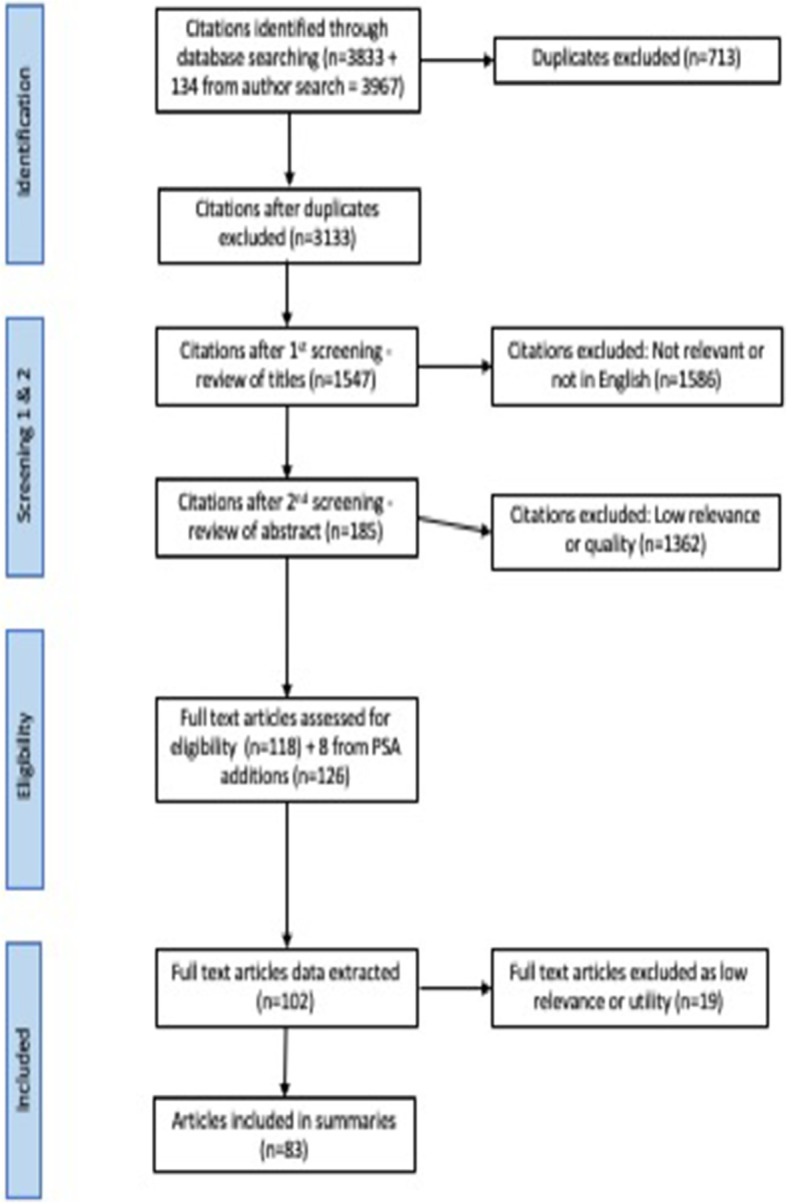
Flow diagram detailing the search process

We consulted four key databases: Scopus, CINAHL, Medline (including Cochrane Reviews), and PsycINFO to retrieve references. Our key inclusion criteria were: books, book chapters, journal articles and systematic reviews on regulation relating to all regulated professions covered by the PSA published in English since the start of 2011; such literature must be linked to specific regulatory functions (including fitness to practise, standard setting, quality assurance etc). The full search strategy complete with Boolean strings is reported in Appendix 1. We retrieved 3833 records, which reduced to 3179 after removal of duplicates. We added a further 134 publications from a search of key authors known to be active in the field, removing 59 duplicates. The final list contained 3133 records and the full search process is detailed in Fig 1.

Because of the large number of records retrieved, we used a four-step process of screening.

1. Review titles, excluding those which clearly had no relevance to the study (not a regulated profession, no reference to regulation) or were not in English; 1586 papers were excluded at this stage
2. Review abstracts, excluding those published before 2011, commentaries, editorials and opinion pieces, and those which lacked clear research question(s), or offered insufficient quality evidence to answer research questions. To determine quality, the records were divided into rough sections and individual members of the team reviewed sections, rating them on a scale of 1–5 (1 being lowest) to indicate scientific quality and relevance to the study’s aim; we then exchanged our sections and reviewed each other’s work. 1362 papers rated 3 or less were excluded at this stage. Where there was significant disagreement between reviewers, these were discussed and rated by the wider team.
3. Review full papers. The 185 papers remaining were sorted into two groups depending on the star ratings we had previously allocated plus the degree of relevance to the study’s key aim, which was to inform the work of regulators in commissioning, analysing, interpreting and using research data to support policy development and implementation. We set the less relevant papers to one side, designating this the ‘out group’; these papers were not completely lacking in relevance but offered little or no practical application to regulators’ work. The ‘out group’ was retained in case future work is needed.
4. The remaining 118 papers were designated the ‘in group’ and to these were added 8 very recently published papers that had been presented at a PSA conference. These were analysed in detail by all members of the research team. A further 35 papers were reallocated to the ‘out group’, leaving 83 publications we assessed as having greatest relevance. These comprise the core literature underpinning the REA. We were assisted in our definition of relevance throughout the screening process by the PSA’s own definition of the four roles of the regulator [6]. During the process the team discovered a number of papers that did not fit easily into any of these four categories since their focus was either on the wider regulatory context within which regulators work or on the broader impact of regulatory guidance on the professions. After careful consideration we added these as additional themes.

i. Harm prevention and patient safety – evidence concerning regulatory activity that aims to reduce the number of patient-safety incidents and to identify and prevent adverse events before they occur.
ii. Fitness to practise, misconduct, complaints and disciplinary procedures – evidence relating to the processes by which regulators deal with complaints or concerns about registered practitioners.
iii. Quality assurance of education and training – evidence concerning how regulators work to ensure that graduates of education and training courses are properly prepared to practise as registered professionals. This includes the work done by regulators in setting standards for, approving, inspecting and ensuring the quality of education and training programmes.
iv. Registration and the maintenance of registers – evidence around regulators’ work to ensure the accuracy and recentness of the public registers of practitioners who are statutorily approved and qualified to practise as health and care professionals in the UK.
v. Guidelines and standards – how regulatory guidance is modified, interpreted and applied, the impact of standards on practice and training, the impact of government and employer regulations on regulatory guidance (e.g. ethical practice, working hours, workforce planning etc.).
vi. Relations with regulatory bodies – research and reports commissioned by regulatory bodies; practitioners’ attitudes to and reception of regulatory guidance.

The final ‘in group’ studied comprised a diverse collection of evaluation and impact studies and other, more general, articles. The evaluation and impact studies rarely used experimental designs, with the methods usually based on the interpretation of previously collected data, but all clearly addressed issues regarding the impact, effect, evaluation, or outcomes of regulatory activity. The general publications mainly provided overviews, summaries of the current position or debates, but relevance was assured by their focus on the effects of regulation.

## Results

We report key findings from our content analysis within each of our final six thematic groupings plus the website review. Table 1 gives an overview of the total number of texts included in the final analysis by theme, source country of data and professions covered.

**Table 1:** Number of papers reviewed, by theme, origin of data, professional groups and study methods

Theme	Total number of texts included in the REA	Source countries for data	Professional groups	Methods
Harm prevention and patient safety	8	UK (6) Australia (1) Canada (1)	Non-specific (4) Doctors (2) Nurses (1) Pharmacists (1)	Interviews (4) Mixed methods (3) Secondary data analysis (1)
FtP, misconduct, complaints and disciplinary measures	22	Australia (6) Canada (1) UK (15)	Doctors (6) Multiple (5) Midwives & nurses (4) Dentists (3) Non-specific (2) Social workers (1) Paramedics (1)	Secondary data analysis (11) Mixed methods (4) Interviews (2) Survey (2) Document analysis (2) Policy review (1)
Education and training	16	UK (4) Ireland (4) Canada (4) Multiple (3) Australia (1)	Doctors (3) Nurses (2) Paramedics (2) Dentists (2) Multiple (2) Chiropractors (1) Radiographers (1) Others (3)	Interviews (4) Survey (2) Mixed methods (2) Document analysis (1) Secondary data analysis (1)
Guidelines and standards	11	UK (9) Netherlands (1) Sweden (1)	Midwives & nurses (9) Doctors (2)	Surveys (4) Interviews (3) Secondary data analysis (2) Discussion paper (2)
Registration and maintenance of registration	10	UK (5) Australia (4) Canada (1)	Midwives & nurses (5) Doctors (4) Social workers (1)	Interviews (4) Surveys (2) Mixed methods (2) Document analysis (1) Secondary data analysis (1)
Relations with the regulatory body	14	Canada (6) UK (4) Australia (2) Norway (1) Multiple (Europe) (1)	Multiple (4) Midwives & nurses (4) Doctors (2) Social workers (1) Others (3)	Interviews (4) Mixed methods (4) Document analysis (4) Survey (2)
**Total**	**81**			

### Harm prevention and patient safety

We considered eight papers within this theme. Although numbers in this section were small, this may be due to difficulties in isolating this specific theme from related themes, such as misconduct or fitness to practise. The focus of the papers in this group was primarily on new regulatory models that specifically aimed to improve patient safety in secondary care settings. All papers reported a number of conceptual and practical issues concerning how quality of care is assessed and measured. For example, aspects that support implementation of new programmes and policy instruments include allowing time to understand changes, training and sharing data from previous evaluation exercises. In contrast, teams that lack expertise in handling and understanding evaluation data experience difficulties in interpreting effects and implementing effective solutions [11, 12]. One paper, however, suggested that at least one tool based on statistical data, often seen as a cheaper option for prioritising inspection visits, may itself be flawed and that surveillance tools based on statistical data may be untrustworthy [13]. Because inspections create tensions, stable and committed teams of inspectors work more effectively and harmoniously than temporary teams created for a single short-term purpose [14].

Leaders need flexibility and tolerance to manage risks effectively and to achieve compliance; this advice also applies to regulators. Beaussier et al. [15] studying the impact of risk-based policy instruments, argue that unclear goals and inflexible implementation may lead to the failure of initiatives to improve the quality, effectiveness, proportionality and legitimacy of healthcare regulation [16]. Moreover, practice standards and rigid protocols imposed without simultaneous clarity around goals and flexibility to respond to dangerous circumstances will push the management of risk on to individuals [17]. Patient safety could be enhanced by a common system of language assessment.

### Fitness to practise, complaints, misconduct and disciplinary measures

We studied 22 papers within this theme. Papers in this section mainly comprised quantitative studies with most reporting secondary analyses of existing data. In general, they were analyses of fitness to practise (FtP) cases. Where studies involved collecting and analysing primary data, these usually reported a mixed methods approach such as the qualitative analysis of interview or focus group data to inform the results of quantitative questionnaires.

Evidence shows that very few professionals undergo fitness to practise procedures; moreover, there is some evidence to suggest that relatively small numbers of practitioners may be responsible for the majority of complaints [18]. Within these small numbers, however, there is clear over-representation of certain demographics and professions: black and minority ethnic groups [19], older males, [20], overseas-trained [21], dentists and doctors, [20, 22–24], chiropractors [25] social workers [26] and paramedics [27]. Nurses were underrepresented in referrals but if the complaint was upheld, were more likely to receive severe penalties [20]. Tiffin et al. also showed evidence that language proficiency is associated with over-representation of certain groups in referrals [28].

Various reasons for this apparent inequity in patterns of complaints were offered: lack of diversity in investigating panels [19], stressful and competitive environments and resource deficiencies [27], failures in record keeping [22] and cultures that focus on blame and penalties rather than support and remediation [27]. Moreover, there may be significant variations in judgement outcomes at all levels both within single professions across jurisdictions [29], and also between professions such as doctors, nurses and allied health professions [30] even where the trigger complaint was the same [29].

The most common causes of complaints to regulators are shared by all professions, and are usually related to deficiencies in clinical care, such as errors in treatment or substance misuse. Other key triggers include poor communication, unprofessional behaviour and interpersonal relationships [30]. While different professions may show varying proportions of these complaints, this may be because recording methods and coding taxonomies not only vary between professions but may not be nuanced enough to capture the full spectrum of issues [30, 31].

Studies reveal that social and environmental dimensions may also have an effect on types of misconduct reported. These factors include: demanding and unsupportive work environments, competition and stress, and a culture that focuses on individual blame instead of learning. Highly competitive environments may lead to a higher proportion of complaints from fellow practitioners [26].

Fitness to practise mechanisms or decisions are often unclear and slow to progress for individuals and organisations and this may create unnecessary stress. The psychological distress resulting from FtP investigations may lead to worsening patient care, mental health concerns [32] and even suicide [33]. Failure to disclose disability or ill health may affect FtP; while disclosure may benefit practitioners if it leads to appropriate workplace adaptations, many were reluctant to reveal special needs because of fears around stigmatisation [34]. Concerns about damage to reputations and loss of business may also affect incident reporting [35] and there is significant anxiety regarding vexatious complaints [32] .

Almost all studies indicate support for clearer guidance, greater harmonisation and standardisation of FtP processes both within and between professions, more support for professionals subject to FtP procedures and a quicker and more streamlined approach to complaint resolution. They also stress that more data are needed to gain a more complete picture. Many of these papers’ conclusions were anticipated in *Promoting Professionalism, Reforming Regulation* [36] which concluded that if these things were to be achieved in the UK, then current legislation would need to be changed to give more autonomy to professional regulatory bodies.

### Education and training

The 16 papers in this group address both undergraduate education and continuing professional development (CPD). For ease of analysis we divided the papers into two main groups: those concerned with the setting of standards in higher education, including reviews and evaluations of curricula; and those relating to post-qualification training and CPD.

Generally, studies in the first group call for increased standardisation, coordinated efforts and greater collaboration both with regards to within-profession learning and teaching, and also more widely [37]. Regulators’ distinctive cultures around patient safety present a potential barrier to curricular integration [38, 39]. Standardisation and harmonisation of regulators’ quality assurance processes, particularly of clinical experience were seen as key factors in ensuring good educational experiences [40, 41]. Lack of sufficient financial incentives and difficulties securing clinical placements may hinder efforts to implement the practical application of learning [40].

Studies stress that context is a crucial consideration if education reforms on curricula or CPD programmes are to be implemented successfully. Intra-professional learning depends on positive attitudes among faculty, but evidence is lacking concerning patients and carers’ participation, especially in clinical assessments. Internal politics may influence which curricula or programmes are chosen and implemented.

Many professions have increased the academic content of their education and training, and there are mixed reports on the effects of this depending on the profession concerned. In the case of nursing, increased academisation has been seen as a positive change. Two studies expressed tensions as new curricula gave rise to accusations that regulators were less interested in practical experience than in academic knowledge. This created some friction between more recent graduates and those who had been trained in earlier, apprenticeship approaches within both paramedic [42] and chiropractic education [43].

The second group of papers reported that continuing professional development (CPD) was generally appreciated and valued [44–47]. CPD is now increasingly widespread and mandatory in the health and care professions [48]. There is some concern that linking mandatory CPD to remediation may be perceived as an imposition and paradoxically ‘de-professionalising’ [49]. However, given the arguments in favour of extending lifelong learning within the healthcare professions, it seems as if there will continue to be growth in regulatory requirements for CPD. Moreover, there is scope to increase both the intellectual challenge and the practical application of learning in current CPD programmes [50].

### Guidelines and standards

The group comprised 11 papers analysing and commenting on how guidelines function in practice along with discussing and reporting the effects of changes to guidelines. The majority of publications were from 2013 and 2015 with none postdating 2017.

A key concern of five of the papers in this group was the statutory supervision of midwives. Prior to a King’s Fund review of midwifery regulation [51], UK midwives were regulated by both the Nursing and Midwifery Council (NMC) and local authorities, but this arrangement ceased in 2015 [52–55]. These papers, which mostly addressed the practical issues around how the change was being implemented and how midwives were responding, largely identified areas for improvement but reported that overall, UK midwives supported the new measures and found supervision valuable for professional support.

The remaining studies dealt with the more general impact of changes in guidelines. Some specifically explored the implementation process. There is strong consensus that clarity is essential to the successful implementation of change but that this is sometimes lacking [56, 57]. In order to support guideline changes, a variety of new strategies or instruments must be considered. This is a particular issue where new, hybrid or specialist roles are developed that are not adequately covered by current regulatory guidance [58, 59] and where scope of practice and the associated decision-making frameworks, particularly with respect to care for patients, are not clearly conceptualised [60, 61].

Studies of doctors suggest that even after changes in guidelines, there may be a degree of continuity and inertia as doctors resist any perceived loss of autonomy [59]. One mechanism that may encourage the adoption of new quality improvement measures is competition [62].

### Registration and maintenance of registration

There was a general consensus that processes can be ‘controversial’ within the ten papers that comprised this theme. A number of challenges to registration procedures were revealed. Excessive bureaucracy, technical challenges, inconsistencies, and obstructive gatekeepers combined to make registration processes unpopular with registrants [63, 64]. McGillis Hall et al. also recommend that stakeholders should be involved in the design of licensure exams to improve their appropriateness and sensitivity to different contexts [64].

Likewise, revalidation systems for doctors attracted criticism on the grounds that patients are insufficiently involved [65–67]. Doctors also highlight the tensions caused by conflicting discourses that depict revalidation as an aspect of professionalism and those which suggest that it is about ‘catching bad doctors’ [67, 68]. Bryce et al. drew attention to one of the unfortunate effects of this tension, by which responsible officers were seen to have formed a new governance elite rather than defending doctors’ autonomy with the associated perception that regulation is invading the organizational sphere [68].

This theme also reflected some concerns about areas where guidance from regulators (in this case, regarding midwives’ maintenance of registration) lacks clarity or is not aligned to what practitioners consider best practice [69, 70]. This may cause individual practitioners some concerns about how to make personal choices regarding their own training and registration. In such circumstances they may rely on personal connections and attitudes to CPD to guide their choices [69]. The same authors, in a subsequent study [71], found that many midwives had opted to maintain dual nursing and midwife registrations, making the revalidation process more complicated.

### Relations with the regulatory body

Within this theme, 14 papers were considered. These studies mainly related to relationships between the regulator and registrants. Papers exploring the relationship between the regulator and other institutions (such as academic institutions and employers) were also included in this theme, along with papers relating to relations with governments and the public.

Despite some relationship difficulties, most papers reported a general acceptance of the important role that regulation plays in terms of enhanced standards of practice, public safety and the reassurance and trust that regulation confers on professions [72, 73]. This positive perspective is lent support by two papers studying professions not yet regulated, naturopathy [74] and massage therapy, which conclude that regulation would benefit public, practitioners and profession alike [75].

There was, however, a significant thread of negativity within this theme. Studies reported the views of registrants and other stakeholders who perceived regulators as remote, mistrusted, punitive and unsupportive. One unfortunate effect of this is that professionals may practise defensively [55, 76]. A number of these papers call for regulatory reform, arguing for less complexity and bureaucracy coupled with a more standardised regulatory approach and better collaboration between regulators [77]. However, the continued presence of traditional hierarchies in healthcare continues to threaten the development of more common approaches to regulation [78–80]. Negative attitudes can be exacerbated by evidence of inconsistent practice across regulators, regions or countries. These inconsistencies may present a challenge to workforce mobility, patient and client safety and quality of care [4, 81].

Multiple challenges to implementing reform are noted by the papers in this theme. Authors conclude that regulators planning regulatory changes must ensure that there is greater consultation and engagement with practitioners, patients and other stakeholders [82, 83].

### Website review

We also undertook a review of the websites of the ten regulatory bodies overseen by the PSA Because of the enormous scale and scope of these websites, we focused in particular on the regulators’ annual reports that were current in early 2020. This allowed us a clear point of comparison between all the regulators. We analysed each report thematically, identifying seven key themes: harm prevention, quality assurance of education and training, registration and maintenance of registers, patient safety/care improvement, standard setting and revalidation/CPD. Fitness to practise is the chief preoccupation of the regulators and this was reflected in the annual reports; it was in the top two most frequent themes in every report, closely followed by standard setting and patient safety. This finding needs contextualising, however. Several regulatory bodies used their annual reports to express frustration with outdated legislation around fitness to practise. They took the opportunity to express dissatisfaction with the current, legally-based system which is predicated on punishment and assignment of blame rather than on prevention. An example is the General Dental Council’s 2018 annual report which stated ([84] p9).

*Regrettably, our ability to realise the full potential of a modern, principles-based system of regulation is hampered by what remains an antiquated legislative framework.*

The exception to this preoccupation with FtP within the annual reports was the newly established Social Work England’s annual report 2018/19 [85]; this may have been because the main part of the document was devoted to describing the structure of the new regulator. The other regulators outlined the various measures they were introducing to reduce the number, complexity and burden of FtP procedures including new threshold criteria, increased recruitment of specialist staff, and new measures to close down cases early where this was indicated. Several regulators also committed to measures to reduce the distress caused by lengthy proceedings and providing extra support for registrants. Recent high-profile cases were commonly discussed, with regulators reporting how they were addressing the underlying issues.

### Limitations

We made the decision to use rapid evidence assessment methods instead of a systematic review for two main reasons: the first was that the scope of the project was so wide that using a pre-set protocol might not have produced the breadth of results required; and the second was a pragmatic one; systematic reviews usually take well over a year [86] but because regulation is a highly dynamic field, we wanted to produce results more quickly.

As reported in our methods, our initial searches produced an enormous number of results. A relatively simple institutional search for “nursing” and “professional regulation” produced over 200,000 results. The complexity and scale of the task was increased because of the relatively open research questions, the nine-year timespan, the dozens of professional groups involved, and the international scope of the work. Our work was commissioned by the PSA, which was particularly interested in research relating to its own regulators in UK. While our remit was to consider as wide a range of international literature as practicable, the huge number of results meant we had to make the strategic decision to concentrate on those regions and administrations whose healthcare and regulatory systems are most comparable to those of the UK.

We were able to focus our searches more effectively thanks to a rigorous specialist-designed search strategy involving lengthy and comprehensive search strings. Despite this, we still retrieved extremely high numbers of titles – far too many for us to be able to claim conclusively that all relevant papers had been included and all non-relevant papers excluded. In an attempt to decrease the risks, the research team devised a novel four-stage process to select the most relevant and highest quality papers for review. Additionally, we set to one side a large number of lower quality, or less relevant papers that we deemed to be beyond the scope of the study but that might have some potential relevance to future research.

## Discussion

In discussing these results, we identify several significant challenges for the study of health and care professions regulation that, if addressed, could improve regulators’ access to high quality research evidence. First among these is that our broad search of the academic literature on regulation confirms the 2011 report’s view that the majority of papers report small, uni-professional projects that are local in scope and often appear to be driven by particular agendas. Methods were largely qualitative interviews, surveys or secondary analysis of routinely gathered data such as progression and assessment results or annual regulators’ surveys. Descriptive findings predominated, and we found very few studies that attempted to link findings to effect or causation. There was a significant mismatch between (a) what the regulators’ websites and annual reports had indicated were important research priorities and (b) what researchers were actually publishing. Conclusions frequently reflected a generalised dissatisfaction with and resistance to regulatory changes. These results are unsurprising in view of the predominance of methods aimed to establish the perceptions of practitioners; while practitioners’ perspectives are vitally important to the success of improvement initiatives, there was far less multi-methods research aimed at reliably exploring more generalisable causal linkages between professional regulation and professional behaviour. Consequently, many studies raised concerns without being able to offer sufficiently robust evidence on which a regulator might be able to base a plan to address such concerns.

A further issue with the academic literature on regulation is that it is diffuse; there is one specialist journal (on nursing regulation) but there are no multi-professional journals of healthcare regulation. Whilst numerous researchers are active in the field, healthcare regulation has yet to achieve the status of a recognised academic discipline, unlike commercial, legal and financial regulation, which all have their own academic departments, journals and personal chairs. This is not only a challenge when it comes to locating a body of evidence in the field, but it represents challenges for sustained, programmatic research and also hampers regulators’ efforts to locate and collaborate with experienced academic research teams to explore solutions that would be useful to them.

While regulators are generating and commissioning research that can inform regulatory practice, their budgets are generally limited. It is more common for published research papers to be ‘unfunded’, possibly generated as part of a course of individual postgraduate study rather than the product of a programme of research; this is a probable reason for the very small scale and lack of generalisability of many of the works we retrieved.

## Conclusion

Our work has some important implications for the development of regulation as a field of academic study. While there is no doubt that a great deal of scholarly activity is currently being undertaken relating to the field, it cannot yet be said that health and social care regulation studies has established its place as a recognised academic discipline. While other sectors, such as financial, legal, or aviation studies have their own professoriate, research departments and even specialist journals, health and social care regulation is still developing its own place as an evidence-based field of study and practice. This presents a unique opportunity both for regulators and academics. Regulators, working more closely together, are in a position to set a research agenda for this emergent field by making more effective use of the research literature, refocusing the work of their policy and research departments to ensure that the benefit of engaging with academics and scholarly practice are fully realised, and by ensuring that their research budgets are used more effectively to commission only the highest-quality and most relevant evidence to support their regulatory work, along with targeted dissemination strategies. In return, the academic community could also benefit by making stronger efforts to engage with regulators, treating them less as passive recipients and more as active stakeholders in any research undertaken. We suggest that further work is needed to explore how these deeper engagements between regulators and academic outputs may be created and sustained.

## Supplementary Information

**Additional file 1.**

## Data Availability

Data were collected from publicly available sources. All search menus are included in the appendix to this paper; a list of sources is available from the corresponding author on reasonable request.
