# Supplementary material for: Recent research into healthcare professions regulation: a rapid evidence assessment
Source: BMC Health Serv Res. 2021 Sep 8;21:934. doi: 10.1186/s12913-021-06946-8 (PMC8425088; doi:10.1186/s12913-021-06946-8)
Supplement: Supplementary file 1 — Additional file 1. [file 12913_2021_6946_MOESM1_ESM.docx]

Recent research into healthcare professional regulation: a rapid evidence assessment

Julie Browne (1,2), Alison Bullock (1), Chiara Poletti (1), Dorottya Cserző (1)

1. Cardiff University, Cardiff Unit for Research and Evaluation in Medical and Dental Education (CUREMeDE), 10/12 Museum Place, Cardiff CF10 3BG
2. Cardiff University, Cardiff University School of Medicine, Centre for Medical Education, Neuadd Meirionnydd, Heath Park, Cardiff CF14 4YS

Corresponding author

Julie Browne BA, PGCE, MA, SFHEA, FAcadMEd

Cardiff University School of Medicine, Centre for Medical Education, Neuadd Meirionnydd, Heath Park, Cardiff CF14 4YS

## Appendix 1 – Further Information on Search Strategy

Here is a summary of the queries performed in the different datasets:

1. **Scopus (n=1081, last search 30.01.2020)**

In Scopus, following the exploratory study we developed the following query. With the help of an expert healthcare librarian, we developed a list of search terms for the in-text queries (TITLE-ABS=title and abstract), followed by a limitation based on temporal dimension, geographical, and subject areas.

| 1. **List of professions, as in text terms search**   (TITLE-ABS(orthopt* or physical therapist or arts therapist or biomedical scientist or clinical scientist or hearing aid dispenser or operating department practitioner or nurs* or optom* or dent* or midwif* or pharma* or medica* or osteopath* or chiropract* or social work* or chiropod* or podiatr* or dieti* or occupational therap* or paramedic* or physiotherap* or psycholo* or prosthet* or orthot* or radiograph* or speech and language or doctor* or physician* or general practitioner* or welfare work* or health care profession* or healthcare profession* or health-care profession* or health and care profession*))   1. **Regulation search terms**   AND ((TITLE-ABS(regulat*))   1. **List of policy terms, as in text terms search**   AND ((TITLE-ABS(fitness to practi*)) or (TITLE-ABS(standard setting OR standards of practi*)) or (TITLE-ABS(standard setting OR (standard* W/2 practi**))) or (TITLE-ABS(quality assurance*)) or (TITLE-ABS(regist* W/2 profession*)) or (TITLE-ABS(prevent* W/2 harm*)) or (TITLE-ABS((client* or patient*) W/2 safet*))))  AND  **Geographical limitations**  ( LIMIT-TO ( AFFILCOUNTRY,United States ) OR LIMIT-TO ( AFFILCOUNTRY,United Kingdom ) OR LIMIT-TO ( AFFILCOUNTRY,Australia ) OR LIMIT-TO ( AFFILCOUNTRY,Canada ) OR LIMIT-TO ( AFFILCOUNTRY,Sweden ) OR LIMIT-TO ( AFFILCOUNTRY,Germany ) OR LIMIT-TO ( AFFILCOUNTRY,Netherlands ) OR LIMIT-TO ( AFFILCOUNTRY,Norway ) OR LIMIT-TO ( AFFILCOUNTRY,Switzerland ) OR LIMIT-TO ( AFFILCOUNTRY,France ) OR LIMIT-TO ( AFFILCOUNTRY,Ireland ) ) AND ( LIMIT-TO ( DOCTYPE,ar ) OR LIMIT-TO ( DOCTYPE,re ) )  AND  **Subject area limitations**  ( LIMIT-TO ( SUBJAREA,NURS ) OR LIMIT-TO ( SUBJAREA,MEDI ) OR LIMIT-TO ( SUBJAREA,SOCI ) OR LIMIT-TO ( SUBJAREA,HEAL ) OR LIMIT-TO ( SUBJAREA,PHAR ) OR LIMIT-TO ( SUBJAREA,BUSI ) OR LIMIT-TO ( SUBJAREA,PSYC ) OR LIMIT-TO ( SUBJAREA,MULT ) OR LIMIT-TO ( SUBJAREA,DENT ) )  AND  **Extra limiters:**  **Publication limitations: year and type and language**  ( LIMIT-TO ( PUBYEAR,2020) OR LIMIT-TO ( PUBYEAR,2019) OR LIMIT-TO ( PUBYEAR,2018) OR LIMIT-TO ( PUBYEAR,2017) OR LIMIT-TO ( PUBYEAR,2016) OR LIMIT-TO ( PUBYEAR,2015) OR LIMIT-TO ( PUBYEAR,2014) OR LIMIT-TO ( PUBYEAR,2013) OR LIMIT-TO ( PUBYEAR,2012) OR LIMIT-TO ( PUBYEAR,2011) ) AND ( LIMIT-TO ( LANGUAGE,English ) ) |
| --- |

The dataset reports 1081 documents, which we downloaded in RIS format.

1. **Medline (n=1890** **last search 30.01.2020)**

We accessed MedLine through the portal offered by Ovid. With the help of DM we developed a list of subject headings for professions, policy terms and geographical limitations applicable to this database. When possible, we expanded the subject headings to include lower levels terms.

Exp= subject heading expanded, (TI=title and AB=abstract, in search terms for in text queries )

| **1. List of professions, either as subject heading or in text terms search (all connected by OR)**  1. exp Nurses/  2. nurs*.ti,ab.  3. exp Optometrists/  4. optom*.ti,ab.  5. exp Dentists/  6. dent*.ti,ab.  7. exp Midwifery/  8. midwif*.ti,ab.  9. exp Pharmacists/  10. pharm*.ti,ab.  11. medica*.ti,ab.  12. exp Osteopathic Physicians/  13. osteopath*.ti,ab.  14. exp Chiropractic/  15. chiropract*.ti,ab.  16. exp Social Workers/  17. social work*.ti,ab.  18. exp Podiatry/  19. chiro*.ti,ab.  20. podiatr*.ti,ab.  21. exp Nutritionists/  22. dieti*.ti,ab.  23. exp Occupational Therapists/  24. occupational therap*.ti,ab.  25. exp Allied Health Personnel/  26. paramedic*.ti,ab.  27. exp Physical Therapists/  28. physiotherap*.ti,ab.  29. exp Psychology/  30. psycholo*.ti,ab.  31. prosthet*.ti,ab.  32. orthot*.ti,ab.  33. radiograph*.ti,ab.  34. exp Speech Therapy/ or exp Language Therapy/  35. speech and language.ti,ab.  36. exp Physicians/  37. doctor*.ti,ab.  38. physician*.ti,ab.  39. exp General Practitioners/  40. general practitioner*.ti,ab.  41. welfare work*.ti,ab.  42. exp Health Personnel/  43. health care profession*.ti,ab.  44. healthcare profession*.ti,ab.  45. orthopt*.ti,ab.  46. physical therapist*.ti,ab.  47. arts therapist*.ti,ab.  48. biomedical scientist*.ti,ab.  49. clinical scientist*.ti,ab.  50. hearing aid dispenser*.ti,ab.  51. operating department practitioner*.ti,ab.  **AND**  **2. Regulation search terms(all connected by OR)**  53. Social Control, Formal/  54. regulat*.ti,ab.  **AND**  **3. List of policy terms, either as subject heading or in text terms search (all connected by OR)**  56. fitness to practi*.ti,ab.  57. standard setting.ti,ab.  58. standards of practi*.ti,ab.  59. (standard* adj2 practi*).ti,ab.  60. exp Quality Assurance, Health Care/  61. quality assurance*.ti,ab.  62. (regist* adj2 profession*).ti,ab.  63. (prevent* adj2 harm*).ti,ab.  64. ((client* or patient*) adj2 safet*).ti,ab.  65. exp Patient Safety/  **AND**  **Geographical limitations (all connected by OR)**  69. exp United Kingdom/  70. united kingdom.mp. [mp=title, abstract, heading word, table of contents, key concepts, original title, tests & measures, mesh]  71. uk.mp. [mp=title, abstract, heading word, table of contents, key concepts, original title, tests & measures, mesh]  72. (england or english).mp. [mp=title, abstract, heading word, table of contents, key concepts, original title, tests & measures, mesh]  73. (wales or welsh).mp. [mp=title, abstract, heading word, table of contents, key concepts, original title, tests & measures, mesh]  74. (scotland or scottish).mp. [mp=title, abstract, heading word, table of contents, key concepts, original title, tests & measures, mesh]  75. (irish or ireland).mp. [mp=title, abstract, heading word, table of contents, key concepts, original title, tests & measures, mesh]  76. exp United States/  77. (united states or US or USA or america*).mp. [mp=title, abstract, heading word, table of contents, key concepts, original title, tests & measures, mesh]  78. exp Australia/  79. australia*.mp. [mp=title, abstract, heading word, table of contents, key concepts, original title, tests & measures, mesh]  80. exp Canada/  81. canada*.mp. [mp=title, abstract, heading word, table of contents, key concepts, original title, tests & measures, mesh]  82. exp Sweden/  83. (sweden or swedish).mp. [mp=title, abstract, heading word, table of contents, key concepts, original title, tests & measures, mesh]  84. exp Germany/  85. german*.mp. [mp=title, abstract, heading word, table of contents, key concepts, original title, tests & measures, mesh]  86. exp Netherlands/  87. (netherland* or holland or dutch).mp. [mp=title, abstract, heading word, table of contents, key concepts, original title, tests & measures, mesh]  88. exp Norway/  89. (norway or norwegian*).mp. [mp=title, abstract, heading word, table of contents, key concepts, original title, tests & measures, mesh]  90. exp Switzerland/  91. (swiss or switzerland).mp. [mp=title, abstract, heading word, table of contents, key concepts, original title, tests & measures, mesh]  92. exp France/  93. (french or france).mp. [mp=title, abstract, heading word, table of contents, key concepts, original title, tests & measures, mesh]  **AND**  **Extra limiters:**  **Publication limitations: year and type and language (connected by AND)**  limit 67 to yr=2011 -Current  limit 95 to english language |
| --- |

The results of this query produced 1890 documents

1. **PsycInfo (n=515, last search 30.01.2020)**

We accessed PsycInfo through the portal offered by Ovid. Initially we ran the same Medline query list but found that subject headings do not coincide between the two databases. For this reason, using PsycInfo’s indexation we selected a number of equivalent subject headings to the ones found in MedLine. In particular:

1)The subject heading 'Osteopathic Physicians' was reported invalid in the database. // Instead we included the subject heading ‘Osteopathic medicine’. (Definition from the database: A system of therapy and medicine based on the theory that diseases are chiefly due to a loss of structural integrity, which can be restored by manipulation of the skeleton and muscles).

2) The subject heading 'Chiropractic' was reported invalid in the database. Since nothing else was found, we removed it from the subject line list of the search.

3) The subject heading 'Podiatry' was reported invalid in the database. Since nothing else was found, we removed it from the subject line list of the search.

4) The subject heading 'Nutritionists' was reported invalid in the database. The only possible replacement was nutrition: and we did not include it.

5) ‘Social control, Formal’ was reported invalid in the database. We replaced it with the subject heading ‘Social control’. (Definition from the database: Power of institutions, organizations, or laws of society to influence or regulate behavior or attitudes of groups or individuals. Consider POWER to access references that describe the control an individual has over other persons.)

6) The subject heading 'Quality Assurance, Health Care' was reported invalid in the database. We replaced it with the subject heading Quality of care. (Definition from the database: Quality of medical or mental health care.) Also: we include a broader term; ‘health care delivery’ (Definition from the database: Practices, policies, or referral processes that contribute to making mental and/or medical healthcare personnel, services, or facilities available to persons in need of such care.)

And the related term clinical governance. (Definition from the database: Structure or guidelines most commonly used by the United Kingdom's National Health Service (NHS) to measure, monitor, and improve the quality of health care).

7) Standard settings, which in MedLine was not present as subject heading, was associated with the subject heading Professional Standards. ( Definition from the database: Minimally acceptable levels of quality professional care or services maintained in order to promote the welfare of those who make use of such services.)

8) In PsycInfo there are no subject heading for countries in this database, so we limited the research with the in-text search of geographical terms.

We included temporal and language limitations.

Here is the query list of terms. Exp= subject heading expanded, in search terms for in text queries (TI=title and AB=abstract)

| **List of professions, either as subject heading or in text terms search (all connected by OR)**  1. exp Nurses/  2. nurs*.ti,ab.  3. exp Optometrists/  4. optom*.ti,ab.  5. exp Dentists/  6. dent*.ti,ab.  7. exp Midwifery/  8. midwif*.ti,ab.  9. exp Pharmacists/  10. pharm*.ti,ab.  11. medica*.ti,ab.  12. exp Osteopathic Medicine/  13. osteopath*.ti,ab.  14. chiropract*.ti,ab.  15. exp Social Workers/  16. social work*.ti,ab.  17. chiro*.ti,ab.  18. podiatr*.ti,ab.  19. dieti*.ti,ab.  20. exp Occupational Therapists/  21. occupational therap*.ti,ab.  22. exp Allied Health Personnel/  23. paramedic*.ti,ab.  24. exp Physical Therapists/  25. physiotherap*.ti,ab.  26. exp Psychology/  27. psycholo*.ti,ab.  28. prosthet*.ti,ab.  29. orthot*.ti,ab.  30. radiograph*.ti,ab.  31. exp Speech Therapy/ or exp Language Therapy/  32. speech and language.ti,ab.  33. exp Physicians/  34. doctor*.ti,ab.  35. physician*.ti,ab.  36. exp General Practitioners/  37. general practitioner*.ti,ab.  38. welfare work*.ti,ab.  39. exp Health Personnel/  40. health care profession*.ti,ab.  41. healthcare profession*.ti,ab.  42. orthopt*.ti,ab.  43. physical therapist*.ti,ab.  44. arts therapist*.ti,ab.  45. biomedical scientist*.ti,ab.  46. clinical scientist*.ti,ab.  47. hearing aid dispenser*.ti,ab.  48. operating department practitioner*.ti,ab.  AND  **Regulation search terms (all connected by OR)**  50. exp Social Control/  51. regulat*.ti,ab.  AND  **List of policy terms, either as subject heading or in text terms search (all connected by OR)**  53. fitness to practi*.ti,ab.  54. standard setting.ti,ab.  55. exp Professional Standards/  56. standards of practi*.ti,ab.  57. (standard* adj2 practi*).ti,ab.  58. exp Quality of care/  59. exp Health Care Delivery/  60. exp Clinical Governance/  61. quality assurance*.ti,ab.  62. (regist* adj2 profession*).ti,ab.  63. (prevent* adj2 harm*).ti,ab.  64. ((client* or patient*) adj2 safet*).ti,ab.  65. exp Patient Safety/  AND  **Geographical limitations (all connected by OR)**  67. united kingdom.mp. [mp=title, abstract, heading word, table of contents, key concepts, original title, tests & measures, mesh]  68. uk.mp. [mp=title, abstract, heading word, table of contents, key concepts, original title, tests & measures, mesh]  69. (england or english).mp. [mp=title, abstract, heading word, table of contents, key concepts, original title, tests & measures, mesh]  70. (wales or welsh).mp. [mp=title, abstract, heading word, table of contents, key concepts, original title, tests & measures, mesh]  71. (scotland or scottish).mp. [mp=title, abstract, heading word, table of contents, key concepts, original title, tests & measures, mesh]  72. (irish or ireland).mp. [mp=title, abstract, heading word, table of contents, key concepts, original title, tests & measures, mesh]  73. (united states or US or USA or america*).mp. [mp=title, abstract, heading word, table of contents, key concepts, original title, tests & measures, mesh]  74. australia*.mp. [mp=title, abstract, heading word, table of contents, key concepts, original title, tests & measures, mesh]  75. canada*.mp. [mp=title, abstract, heading word, table of contents, key concepts, original title, tests & measures, mesh]  76. (sweden or swedish).mp. [mp=title, abstract, heading word, table of contents, key concepts, original title, tests & measures, mesh]  77. german*.mp. [mp=title, abstract, heading word, table of contents, key concepts, original title, tests & measures, mesh]  78. (netherland* or holland or dutch).mp. [mp=title, abstract, heading word, table of contents, key concepts, original title, tests & measures, mesh]  79. (norway or norwegian*).mp. [mp=title, abstract, heading word, table of contents, key concepts, original title, tests & measures, mesh]  80. (swiss or switzerland).mp. [mp=title, abstract, heading word, table of contents, key concepts, original title, tests & measures, mesh]  81. (french or france).mp. [mp=title, abstract, heading word, table of contents, key concepts, original title, tests & measures, mesh]  AND  **Extra limiters**  **Publication limitations: year and type and language (connected by AND)**  84. limit 83 to yr=2011 -Current  85. limit 84 to english language |
| --- |

1. **CINAHL (n=353,** **last search 30.01)**

We accessed CINAHL through the portal offered by EBSCO. We selected a list of subject heading from the ones suggested by the portal, exploding the subject heading, i.e. including the lower categories of terms (when possible). To that list we added the Boolean query previously used in SCOPUS and MedLine (including eventual changes in spelling, preferred terms as in the case of physician rather than doctor). We ran the search terms for in-text queries in the title and abstract (TI=title and AB=abstract).

We reduced the number of results by including geographical criteria (i.e. same as in the other queries), type and years of publications (i.e. Journal articles from 2011 until today) and English language.

| 1. **List of professions, either as subject heading or in text terms search (all connected by OR)**   (MH Medical Technologists)  (MH Emergency Medical Technicians)  (MH Health Personnel+)  (MH Physicians+)  (MH Language Therapy)  (MH Speech Therapy+)  (MH Psychologists)  (MH Physical Therapists)  (MH Allied Health Personnel+)  (MH Occupational Therapists)  (MH Nutrition Services+)  (MH Podiatry)  (MH Social Workers)  (MH Chiropractic+)  (MH Osteopaths)  (MH Pharmacists)  (MH Midwifery+)  (MH Dentists+)  (MH Optometrists)  (MH Nurses+)  (MH Radiologic Technologists)  (MH Dietitians)  (MH Surgical Technologists)  (MH Hearing Aid Fitting)  (TI ( Nurs* OR optom* OR dent* OR midwif* OR pharm* OR medica* OR osteopath* OR chiropract* OR social work* OR chiro* OR podiatr* OR nutrition* OR dieti* OR occupational therap* OR Allied Health Personnel OR paramedic* OR Physical Therapists OR physiotherap* OR psycholo* OR prosthet* OR orthot* OR radiograph* OR Speech Therapy OR Language Therapy OR speech and language OR doctor* OR physician* OR general practitioner* OR welfare work* OR Health Personnel OR health care profession* OR healthcare profession* OR orthopt* OR physical therapist* OR arts therapist* OR biomedical scientist* OR clinical scientist* OR hearing aid dispenser* OR operating department practitioner* )  OR  ( AB ( Nurs* OR optom* OR dent* OR midwif* OR pharm* OR medica* OR osteopath* OR chiropract* OR social work* OR chiro* OR podiatr* OR nutrition* OR dieti* OR occupational therap* OR Allied Health Personnel OR paramedic* OR Physical Therapists OR physiotherap* OR psycholo* OR prosthet* OR orthot* OR radiograph* OR Speech Therapy OR Language Therapy OR speech and language OR doctor* OR physician* OR general practitioner* OR welfare work* OR Health Personnel OR health care profession* OR healthcare profession* OR orthopt* OR physical therapist* OR arts therapist* OR biomedical scientist* OR clinical scientist* OR hearing aid dispenser* OR operating department practitioner* ) )  AND   1. **Regulation search terms**   TI regulat* OR AB regulat* OR  (MH Professional Regulation)  AND   1. **List of policy terms, either as subject heading or in text terms search (all connected by OR)**   (MH Patient Safety+)  (MH Harm Reduction)  (MH Professional Recognition)  (MH Quality of Health Care+)  (TI(fitness to practi* OR standard setting OR standards of practi* OR (standard* n2 practi*) OR quality assurance* OR (regist* n2 profession*) OR (prevent* n2 harm*) OR ((client* or patient*) n2 safet*) OR Patient Safety ))  OR (AB ( regulat* OR fitness to practi* OR standard setting OR standards of practi* OR (standard* n2 practi*) OR quality assurance* OR (regist* n2 profession*) OR (prevent* n2 harm*) OR ((client* or patient*) n2 safet*) OR Patient Safety))  AND  **geographical limitation as subject heading (all connected by OR)**  (MH France)  (MH Switzerland)  (MH Norway)  (MH Netherlands)  (MH Germany)  (MH Sweden)  (MH Canada+)  (MH United States+)  (MH United Kingdom+) OR (MH Great Britain+)  **Extra limiters**  Published Date: 20110101-20201231;  English Language;  Exclude MEDLINE records;  Geographic Subset: Australia & New Zealand, Canada, Continental Europe, Europe, UK & Ireland, USA;  Publication Type: Book, Book Chapter, Journal Article, Systematic Review; Language: English |
| --- |
